# Supplementary material for: Comparison of Short-Term Effects of Different Spinal Manipulations in Patients with Chronic Non-Specific Neck Pain: A Randomized Controlled Trial
Source: Healthcare (Basel). 2024 Jul 5;12(13):1348. doi: 10.3390/healthcare12131348 (PMC11241534; doi:10.3390/healthcare12131348)
Supplement: Supplementary file 1 [file healthcare-12-01348-s001.zip › healthcare-3071714-supplementary.docx]

| **Supplementary Table S1**. Pre and post-treatment values in the upper cervical spine manipulation group. | | | | | |
| --- | --- | --- | --- | --- | --- |
| Measures | Baseline | After one week | Within-group change scores | p-value ^a^ | Rosenthal's r |
| **Pain intensity (0 -10 points)** | | |  |  |  |
|  | 4.1 ± 2.3 | 2.2 ± 2.4 | 1.9 (1.4, 2.4) | 0.001 ^†^ | 0.80 |
| **Neck Disability (0-50 points)** | | |  |  |  |
|  | 22.3 ± 5.4 (44.6%) | 19.5 ± 6.8 (38.9%) | 2.8 (1.9, 5.0) (5.7%) | 0.001 ^†^ | 0.16 |
| **Active CROM (degrees)** | | | |  |  |
| Flexion | 52.0 ± 13.0 | 49.9 ± 10.3 | 2.1 (1.3, 4.1) | 0.01 ^†^ | 0.18 |
| Extension | 67.6 ± 12.6 | 65.5 ± 11.9 | 2.1 (1.1, 4.1) | 0.03 ^†^ | 0.17 |
| Right lateral flexion | 36.9 ± 7.6 | 38.8 ± 9.4 | -1.9 (-3.5, -0.1) | 0.01 ^†^ | -0.22 |
| Left lateral flexion | 42.2 ± 9.2 | 43.9 ± 9.0 | -1.7 (-3.1, -0.1) | 0.001 ^†^ | -0.18 |
| Right rotation | 62.4 ± 8.9 | 63.6 ± 8.6 | - 1.2 (-2.8, 0.4) | 0.17 | -0.13 |
| Left rotation | 60.8 ± 11.0 | 67.1 ± 7.8 | - 6.3 (-8.3, -4.5) | 0.001 ^†^ | -0.67 |

Abbreviations: CROM, cervical range of motion.

Values are expressed as mean ± SD for baseline and one week post-treatment means and as mean (95% CI) for within-group change scores.

^a^ p-value obtained by the Wilcoxon test.

^†^  significant difference

| **Supplementary Table S2**. Pre and post-treatment values in the cervicothoracic spine manipulations group. | | | | | |
| --- | --- | --- | --- | --- | --- |
| Measures | Baseline | After one week | Within-group change scores | p-value ^a^ | Rosenthal´r |
| **Pain intensity (0 -10 points)** | | |  |  |  |
|  | 3.7 ± 2.1 | 1.8 ± 2.2 | 1.9 (1.5, 2.4) | 0.001^†^ | 0.88 |
| **Neck Disability (0-50 points)** | | |  |  |  |
|  | 22.7 ± 4.4 (45.4%) | 16.9 ± 3.8 (33.9%) | 5.8 (4.1, 6.7) (11.5%) | 0.001^†^ | 0.77 |
| **Active CROM (degrees)** | |  |  |  |  |
| Flexion | 51.2 ± 12.7 | 51.3 ± 9.7 | -0.1 (-2.2, 2.2) | 0.95 | 0.00 |
| Extension | 68.6 ± 13.9 | 66.5 ± 14.1 | 2.1 (-0.5, 4.6) | 0.15 | 0.15 |
| Right lateral flexion | 38.1 ± 7.7 | 40.8 ± 7.5 | -2.7 (-4.2, -1.2) | 0.001^†^ | -0.36 |
| Left lateral flexion | 43.5 ± 9.1 | 47.5 ± 8.8 | -4.0 (-5.7, -1.8) | 0.001^†^ | -0.44 |
| Right rotation | 64.9 ± 10.5 | 65.6 ± 8.4 | -0.7 (-2.8, 1.4) | 0.41 | 0.00 |
| Left rotation | 62.1 ± 7.3 | 67.2 ± 6.5 | -5.1 (-6.3, -3.5) | 0.001^†^ | -0.73 |

Abbreviations: CROM, cervical range of motion.

Values are expressed as mean ± SD for baseline and one week post-treatment means and as mean (95% CI) for within-group change scores.

^a^ p-value obtained by the Wilcoxon test.

^†^  significant difference.
